# Development and validation of a dynamic nomogram for individualized prediction of survival in patients with colon cancer

**DOI:** 10.1038/s41598-024-78783-8

**Published:** 2024-11-14

**Authors:** Yuting Sun, Longchang Huang, Xiaoming Shen, Zenghui Yang, Binghua Xu, Chuanqing Bao, Yifan Shi

**Affiliations:** 1 Department of Infectious Diseases, Jiangnan University MedicalCenter, 68 Zhongshan Road, Wuxi, 214000 Jiangsu China; 2 Department of Gastrointestinal Surgery, Affiliated Hospital of Jiangnan University, 1000 Hefeng Road, Wuxi, 214000 Jiangsu China

**Keywords:** Colon cancer, Postoperative survival, NRS 2002, Skeletal muscle mass index, Nomogram, Gastroenterology, Risk factors

## Abstract

Current tools for predicting survival outcomes in colon cancer patients predominantly rely on clinical and pathologic characteristics. However, accumulating evidence demonstrates a significant correlation between nutritional status and patient outcomes. This study aimed to establish a new dynamic nomogram for individualized prediction of postoperative overall survival (OS). The clinicopathological and nutritional data of colon cancer patients undergoing radical resection were retrospectively collected and randomly divided into the primary and validation cohorts. Risk factors on OS rates were investigated by Cox analyses and, the nomogram was constructed using significant predictors. Among 1,024 patients, 341 deaths were observed after median follow-up of 54 months. Five independent prognostic factors, including nutritional status assessments, were incorporated into the nomogram. The C-index regarding 1-, 3-, and 5-year OS were 0.830, 0.859, and 0.757 in the primary cohort and 0.843, 0.870, and 0.773 in the validation cohort, respectively. Calibration curves for the probability of OS exhibited an optimal agreement. Decision curve analyses revealed the greater application value of the nomogram than the TNM staging system. Based on the nomogram, patients could be stratified into three scenarios with significant prognostic classification (*P* < 0.0001). In conclusion, we developed and validated an easy-to-use dynamic nomogram for predicting postoperative OS in colon cancer patients.

## Introduction

Colon cancer is a leading cause of cancer-related deaths globally, with about 1.1 million new cases reported annually (5.96% of total cases), significantly impacting patients worldwide^1^. In China, colon cancer ranks second in incidence and fourth in mortality^2^. Although advances in surgery and adjuvant chemotherapy have improved the prognosis of colon cancer^3^, the long-term overall survival (OS) remains unsatisfactory. Over 29% of colon cancer patients will experience mortality within 5 years following appropriate surgery and adjuvant therapy^4,5^. Thus, developing new strategies to reduce cancer mortality and improve patient outcomes is crucial.

Current prediction tools for colon cancer primarily rely on clinical and pathologic characteristics, such as sex, age, carcinoembryonic antigen (CEA), T stage, and positive lymph nodes, to predict survival outcomes^6,7,8^. However, malnutrition is prevalent among colon cancer patients, ranging from 30 to 50%, and has been shown to significantly impact clinical outcomes, including increased postoperative complications, prolonged hospital stays, and elevated cancer mortality^9,10,11^. It is widely accepted that malnutrition indirectly accounts for at least 20% death of all cancer patients^12^. Recently, a prospective observational study indicated that individualized nutritional support during the hospital stay could reduce mortality and improve the quality of life for cancer patients with high nutritional risk^13^. Moreover, clinical guidelines recommend nutritional screening for all cancer patients, with interventions ideally initiated if malnutrition risk is present^14^.. Given the growing evidence of nutritional status’s significant influence on colon cancer survival, integrating nutritional assessments into current models may enhance prediction performance and then contribute to better assessing cancer outcomes.

The nomogram can generate individualized predictions by integrating a complex statistical model embracing various prognostic factors and has been widely adopted in clinical practice^15^. To improve the likelihood estimates of survival following colon cancer diagnosis and treatment, clinicians could provide additional nutritional interventions to patients who are malnourished or at risk of malnutrition. As such, we sought to establish a simple and useful dynamic nomogram for predicting 1-, 3-, and 5-year OS based on clinical, pathologic, and nutritional status characteristics among patients with colon cancer. The performance of this prediction model was internally validated and the risk stratification of OS for colon cancer patients was determined according to the nomogram score to evaluate its clinical utility.

## Results

### Basic characteristics

A total of 1,553 patients were eligible for inclusion, of whom 173 were excluded, and 83 were lost to follow-up (Supplementary Fig. 1). Finally, the primary cohort included 865 patients with 224 (25.9%) died, while the validation cohort included 432 patients with 117 (27.1%) died. No significant differences in basic characteristics were observed between the two cohorts (Table 1).

**Table 1:** Baseline characteristics of the primary cohort and validation cohort. Continuous variables are presented as means (standard deviations, SDs). Categorical variables are expressed as frequencies (percentages). ^a^ Chi-squared test. ^b^Student’s independent t-test. *aCCI* age-adjusted Charlson Comorbidity Index, *ECOG* eastern cooperative oncology group, *CEA* carcinoembryonic antigen, *NRS 2002* nutrition risk screening 2002, *PG-SGA* patient-generated subjective global assessment, *BMI* body mass index, *LL3MI* low third lumbar spine skeletal muscle index, *NRI*, nutritional risk index, *CCI* comprehensive complication index.

Characteristic	All patients (n = 1297)	Primary cohort (n = 865)	Validation cohort (n = 432)	*P*
**Demographics and clinical features**				
Female, No. (%)	563(43.4)	373(43.1)	190(44.0)	0.768^a^
Age, years, mean (SD)	65.2(9.1)	65.5(8.5)	64.5(10.2)	0.099^b^
Age ≥ 60 years, No. (%)	755(58.2)	511(59.1)	244(56.5)	0.372^a^
BMI, kg/m^2^, No. (%)				
< 18.5	107(8.2)	71(8.2)	36(8.3)	0.748^a^
18.5–24	1008(77.7)	677(67.2)	331(76.6)	
> 24	182(14.0)	117(64.3)	65(15.0)	
aCCI, No. (%)				
0–1	262(20.2)	175(20.2)	87(20.1)	0.888^a^
2–3	569(43.9)	383(44.3)	186(43.1)	
≥ 4	466(35.9)	307(35.5)	159(36.8)	
ECOG, No. (%)				
0	900(69.4)	598(69.1)	302(69.9)	0.775^a^
1–2	397(30.6)	267(30.9)	130(30.1)	
CEA ≥ 5 μg/L, No. (%)	486(37.5)	322(37.2)	164(38.0)	0.796^a^
Hypoproteinemia, No. (%)	469(36.2)	307(35.5)	162(37.5)	0.478^a^
Bowel obstruction, No. (%)	380(29.3)	250(28.9)	130(30.1)	0.657^a^
**Nutritional status**				
NRS 2002, No. (%)				
0–2	668(51.5)	456(52.7)	212(49.1)	0.439^a^
3–4	389(30.0)	251(29.0)	138(31.9)	
≥ 5	240(18.5)	158(18.3)	82(19.0)	
PG-SGA, No. (%)				
A	972(74.9)	652(75.4)	320(74.1)	0.415^a^
B	189(14.6)	129(14.9)	60(13.9)	
C	136(10.5)	84(9.7)	52(12.0)	
LL3MI, No. (%)	500(38.6)	333(38.5)	167(38.7)	0.955^a^
NRI, No. (%)				
≥ 100	723(55.7)	461(53.3)	262(60.6)	0.083^a^
≥ 97.5 to < 100	293(22.6)	207(23.9)	86(19.9)	
≥ 83.5 to < 97.5	180(13.9)	124(14.3)	56(13.0)	
< 83.5	101(7.8)	73(8.4)	28(6.5)	
Total protein, g/L, mean (SD)	73.4(9.0)	73.7(9.1)	72.8(8.8)	0.091^b^
Hemoglobin, g/L, mean (SD)	125.7(13.9)	126.1(14.0)	124.9(13.8)	0.139^b^
Triglycerides, mmol/L, mean (SD)	1.46(0.53)	1.47(0.52)	1.44(0.53)	0.327^b^
Cholesterol, mmol/L, mean (SD)	4.80(1.00)	4.78(0.99)	4.84(1.04)	0.318^b^
**Surgical and staging information**				
Operation type, No. (%)				
Laparoscopy	1052(81.1)	697(80.6)	355(82.2)	0.488^a^
Laparotomy	245(18.9)	168(19.4)	77 (17.8)	
CCI ≥ 26.2, No. (%)	219(16.9)	137(15.8)	82(19.0)	0.154^a^
Tumor location, No. (%)				
Right-sided	513(39.6)	336(38.8)	177(41.0)	0.749^a^
Transverse	93(7.2)	62(7.2)	31(7.2)	
Left-sided	691(53.3)	467(54.0)	224(51.9)	
Tumor size ≥ 5 cm, No. (%)	603(46.5)	394(45.5)	209(48.4)	0.335^a^
Differentiation, No. (%)				
High	79(6.1)	56(6.5)	23(5.3)	0.652^a^
Moderate	576(44.4)	379(43.8)	197(45.6)	
Low	642(49.5)	430(49.7)	212(49.1)	
Histology, No. (%)				
Adenocarcinoma	1101(84.9)	726(83.9)	375(86.8%)	0.173^a^
Non-Adenocarcinoma	196(15.1)	139(16.1)	57(13.2)	
T stage, No. (%)				
T1	242(18.7)	168(19.4)	70(16.2)	0.069^a^
T2	269(20.7)	184(21.3)	79(18.3)	
T3	297(22.9)	184(21.3)	117(27.1)	
T4	489(37.7)	329(38.0)	166(38.4)	
N stage, No. (%)				
N0	692(53.4)	474(54.8)	218(50.5)	0.333^a^
N1	370(28.5)	240(27.7)	130(30.1)	
N2	235(18.1)	151(17.5)	84(19.4)	
TNM stage, No. (%)				
I	320(24.7)	230(26.6)	90(20.8)	0.073^a^
II	372(28.7)	244(28.2)	128(29.6)	
III	605(46.6)	391(45.2)	214(49.5)	
**Outcome**				
Overall survival, No. (%)	956(73.7)	641(74.1)	315(72.9)	0.647^a^
1-year survival, No. (%)	1235(95.2)	826(95.5)	409(94.7)	0.517^a^
3-year survival, No. (%)	1106(85.3)	743(85.9)	363(84.0)	0.371^a^
5-year survival, No. (%)	974(75.1)	654(75.6)	320(74.1)	0.059^a^

## Factor selection for the predictive model

**Fig. 1:**
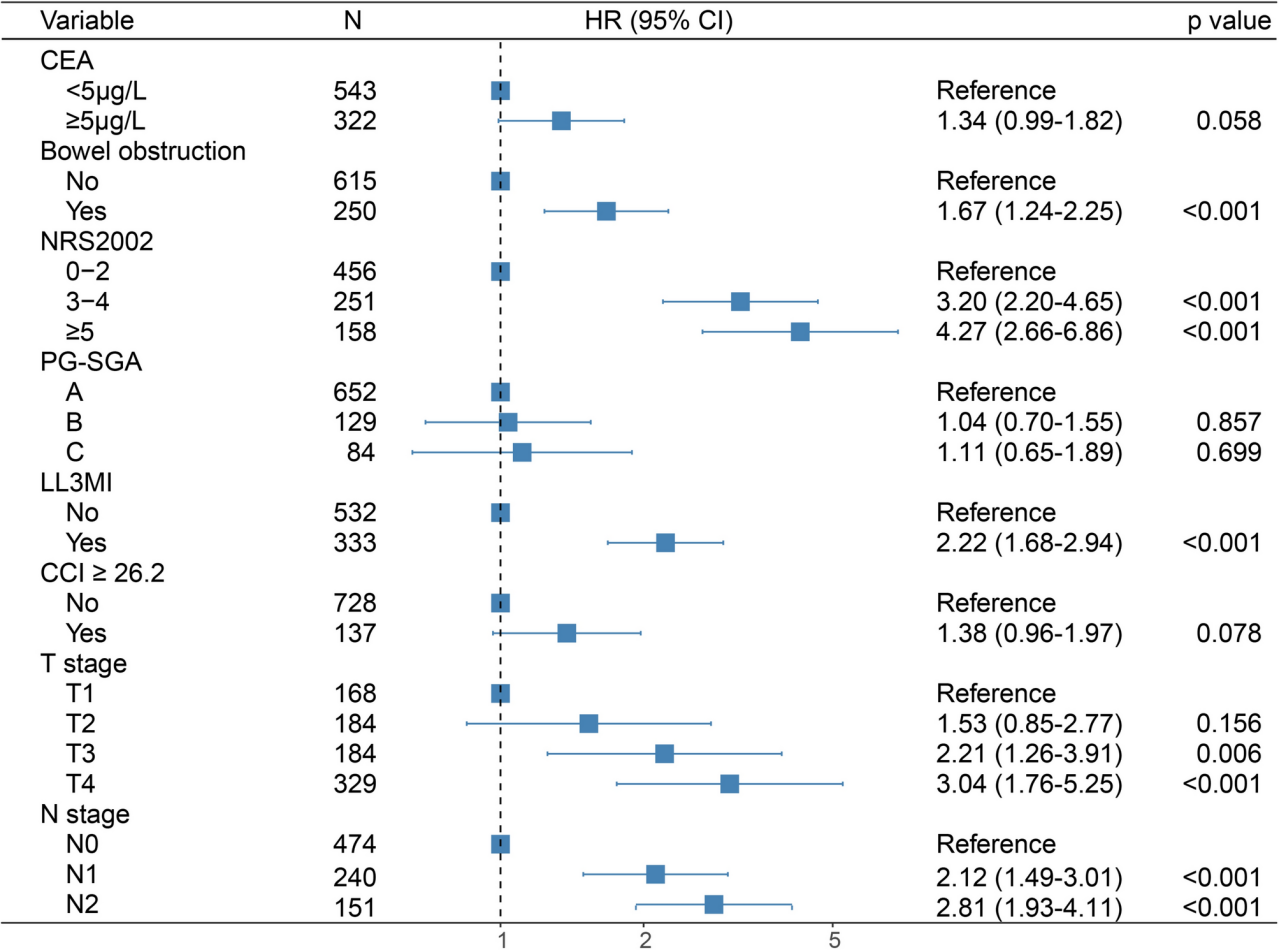
The forest plot of multivariate Cox regression analyses.

Univariate and multivariate analyses were performed in the primary cohort to assess the clinicopathological features and nutritional status, and the results are summarized in Table 2. Five prognostic variables were independently associated with OS. Colon cancer patients with mild and severe nutritional risk had a 3.2-fold and 4.27-fold higher mortality risk than those without nutritional risk, respectively (NRS 2002 3–4 score hazard ratio [HR]: 3.20; 95% CI: 2.20–4.65; *P* < 0.001; NRS 2002 ≥ 5 score HR: 4.27; 95% CI: 2.66–6.86; *P* < 0.001). Death risk in colon cancer patients with LL3MI was 2.22-fold compared to those without LL3MI (HR: 2.22; 95% CI: 1.68–2.94; *P* < 0.001). Patients with bowel obstruction had a significantly higher mortality risk (HR: 1.67; 95% CI: 1.24–2.25; *P* < 0.001), as did those with advanced T stage (T3 HR: 2.21; 95% CI: 1.26–3.91; *P* = 0.006; T4 HR: 3.04; 95% CI: 1.76–5.25; *P* < 0.001), and advanced N stage (N1 HR: 2.12; 95% CI: 1.49–3.01; *P* < 0.001; N2 HR: 2.81; 95% CI: 1.93–4.11; *P* < 0.001) (Fig. 1). Results from univariate and multivariate analyses in the validation cohort are provided in Supplementary Table 1.

**Table 2:** Univariate and multivariate Cox regression analysis of OS in the primary cohort patients. The covariates included in the multivariate analysis were CEA, bowel obstruction, NRS-2002, PG-SGA, L3MI, CCI ≥ 26.2, T stage, and N stage. *HR* hazard ratio, *CI* confidence interval.

Variables	Univariate analysis		Multivariate analysis	
	**HR (95% CI)**	***P***	**HR (95% CI)**	***P***
Sex				
Male vs. Female	0.84(0.65–1.09)	0.191	-	-
Age, years				
≥ 60 vs. < 60	1.15(0.88–1.50)	0.321	-	-
BMI, kg/m^2^				
18.5–24 vs. < 18.5	0.85(0.54–1.32)	0.459	-	-
> 24 vs. < 18.5	0.75(0.43–1.32)	0.317	-	-
aCCI				
2–3 vs. 0–1	1.28(0.87–1.87)	0.214	-	-
≥ 4 vs. 0–1	1.44(0.98–2.12)	0.065	-	-
ECOG				
1–2 vs. 0	1.11(0.84–1.46)	0.456	-	-
CEA, μg/L				
≥ 5 vs. < 5	1.53(1.18–1.99)	0.002	1.34 (0.99–1.82)	0.058
Hypoproteinemia				
Yes vs. No	1.29(0.98–1.68)	0.066	-	-
Bowel obstruction				
Yes vs. No	1.81(1.38–2.37)	< 0.001	1.67 (1.24–2.25)	< 0.001
NRS 2002				
3–4 vs. 0–2	2.95(2.16–4.02)	< 0.001	3.20 (2.20–4.65)	< 0.001
≥ 5 vs. 0–2	3.51(2.47–4.97)	< 0.001	4.27 (2.66–6.86)	< 0.001
PG-SGA				
B vs. A	1.98(1.42–2.76)	< 0.001	1.04 (0.70–1.55)	0.857
C vs. A	2.22(1.50–3.28)	< 0.001	1.11 (0.65–1.89)	0.699
LL3MI				
Yes vs. No	2.81(2.15–3.67)	< 0.001	2.22 (1.68–2.94)	< 0.001
NRI				
≥ 97.5 to < 100 vs. ≥ 100	1.08(0.66–1.76)	0.762	-	-
≥ 83.5 to < 97.5 vs. ≥ 100	1.35(0.93–1.96)	0.110	-	-
< 83.5 vs. ≥ 100	1.20(0.87–1.65)	0.269	-	-
Total protein	1.00(0.99–1.02)	0.659	-	-
Hemoglobin	1.00(0.99–1.01)	0.631	-	-
Triglycerides	1.25(0.88–1.46)	0.075	-	-
Cholesterol	0.99(0.87–1.13)	0.921	-	-
Operation type				
Laparoscopy vs. Laparotomy	0.87(0.63–1.19)	0.376	-	-
CCI ≥ 26.2				
Yes vs. No	1.41(1.01–1.96)	0.045	1.38 (0.96–1.97)	0.078
Tumor location				
Transverse vs. Right-sided	1.06(0.66–1.72)	0.800	-	-
Left-sided vs. Right-sided	0.77(0.58–1.01)	0.056	-	-
Tumor size, cm				
≥ 5 vs. < 5	1.17(0.90–1.53)	0.234	-	-
Differentiation				
Moderate vs. High	1.26(0.68–2.35)	0.464	-	-
Low vs. High	1.37(0.74–2.55)	0.320	-	-
Histology				
Adeno vs. Non-Adeno	0.73(0.53–1.02)	0.062	-	-
T stage				
T2 vs. T1	1.99(1.14–3.48)	0.015	1.53 (0.85–2.77)	0.156
T3 vs. T1	2.41(1.39–4.17)	0.002	2.21 (1.26–3.91)	0.006
T4 vs. T1	3.44(2.09–5.66)	< 0.001	3.04 (1.76–5.25)	< 0.001
N stage				
N1 vs. N0	2.77(2.01–3.83)	< 0.001	2.12 (1.49–3.01)	< 0.001
N2 vs. N0	4.14(2.97–5.77)	< 0.001	2.81 (1.93–4.11)	< 0.001

## Development of nomogram

**Fig. 2:**
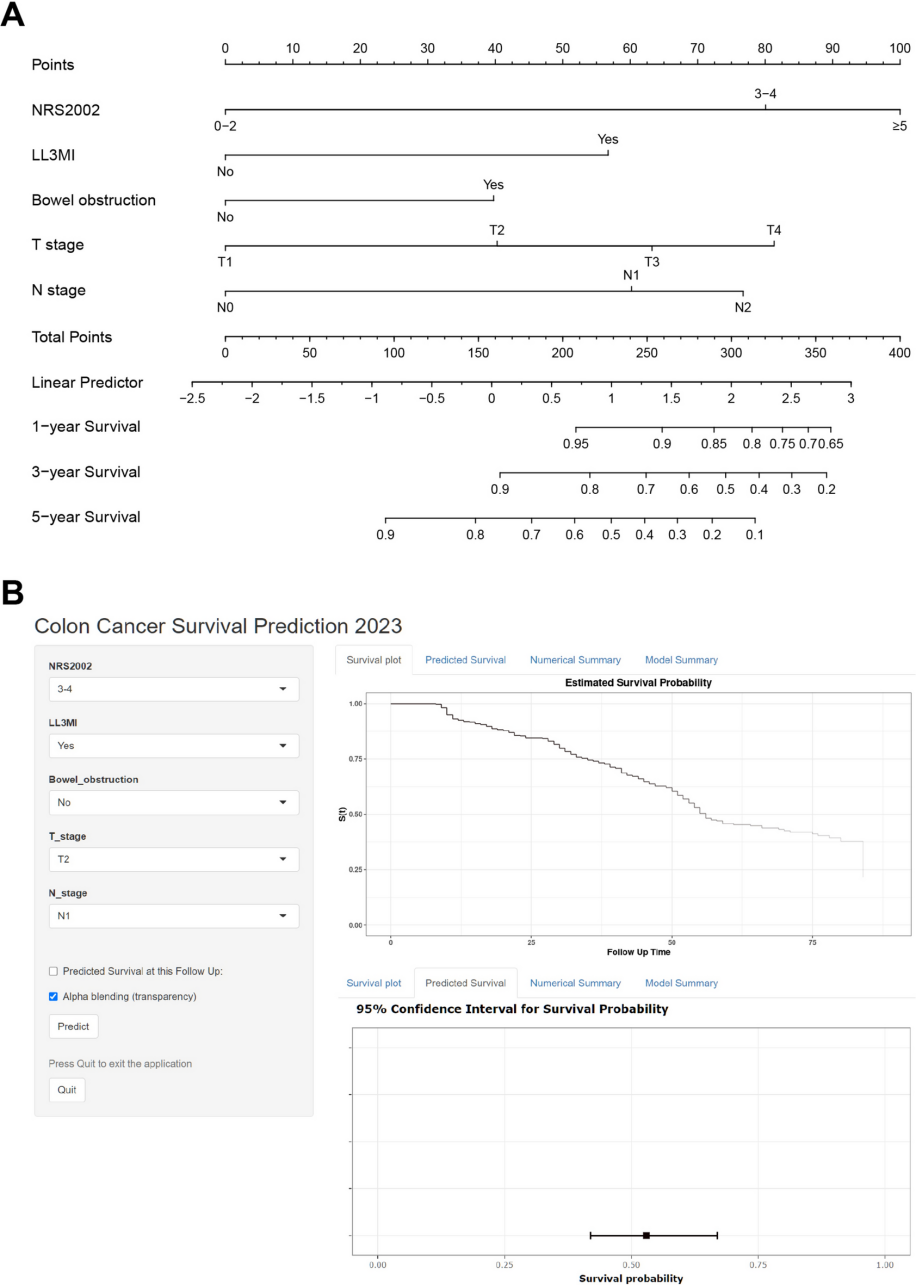
Established nomogram for predicting 1-, 3-, and 5-year OS of colon cancer patients after radical resection by incorporating the five parameters: NRS 2002, LL3MI, bowel obstruction, T stage, and N stage (A). A web-based dynamic nomogram accessible at https://coloncancersurvivalprediction2023.shinyapps.io/DynNomapp/ (B), depicting an example for predicting the postoperative survival probability of a colon cancer patient with NRS 2002 score in the range of 3–4, LL3MI, and T2N1M0.

Using the multivariate Cox model incorporating the independent predictors mentioned above, a simple-to-use nomogram predicting 1-, 3-, and 5-year OS probabilities was developed, as demonstrated in Fig. 2A and is available online (https://coloncancersurvivalprediction2023.shinyapps.io/DynNomapp/) as screenshotted in Fig. 2B. In the nomogram, each factor is assigned a score on the point scale and then added to obtain the total points that correspond to the estimated probability of survival.

## Assessment of prediction model performance

**Fig. 3:**
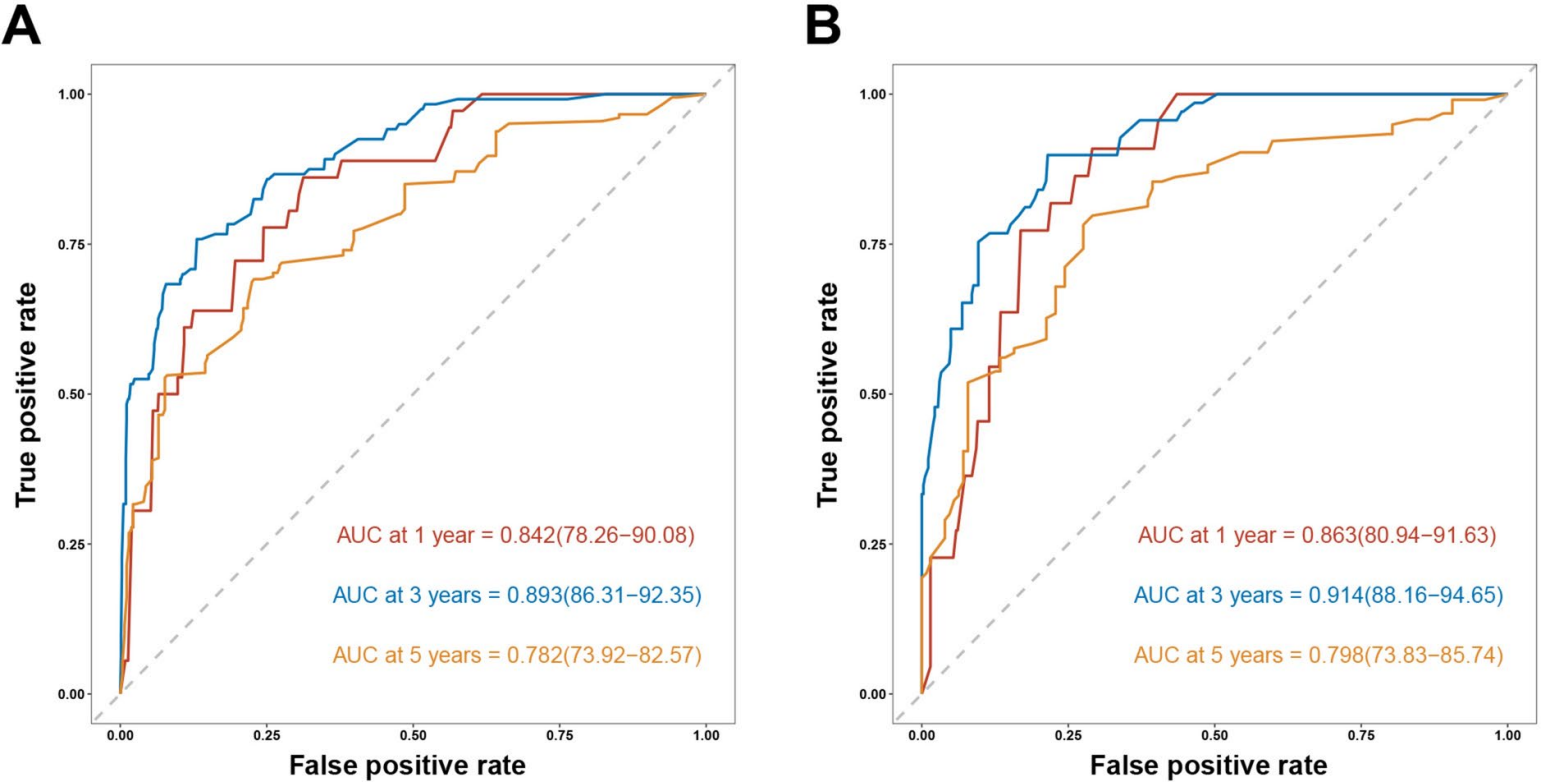
ROC curves of the nomogram model for predicting 1-, 3-, and 5-year OS of colon cancer patients after radical resection in the primary cohort (A) and validation cohort (B).

The performance of the nomogram was evaluated and validated in both cohorts. The ROC curves demonstrated appreciable predictive accuracy of the nomogram regarding 1-, 3-, and 5-year OS rates, with the area under the curve (AUC) reaching 0.842, 0.842, and 0.782 in the primary cohort (Fig. 3A), and 0.863, 0.914, and 0.798 in the validation cohort (Fig. 3B), respectively.

**Fig. 4:**
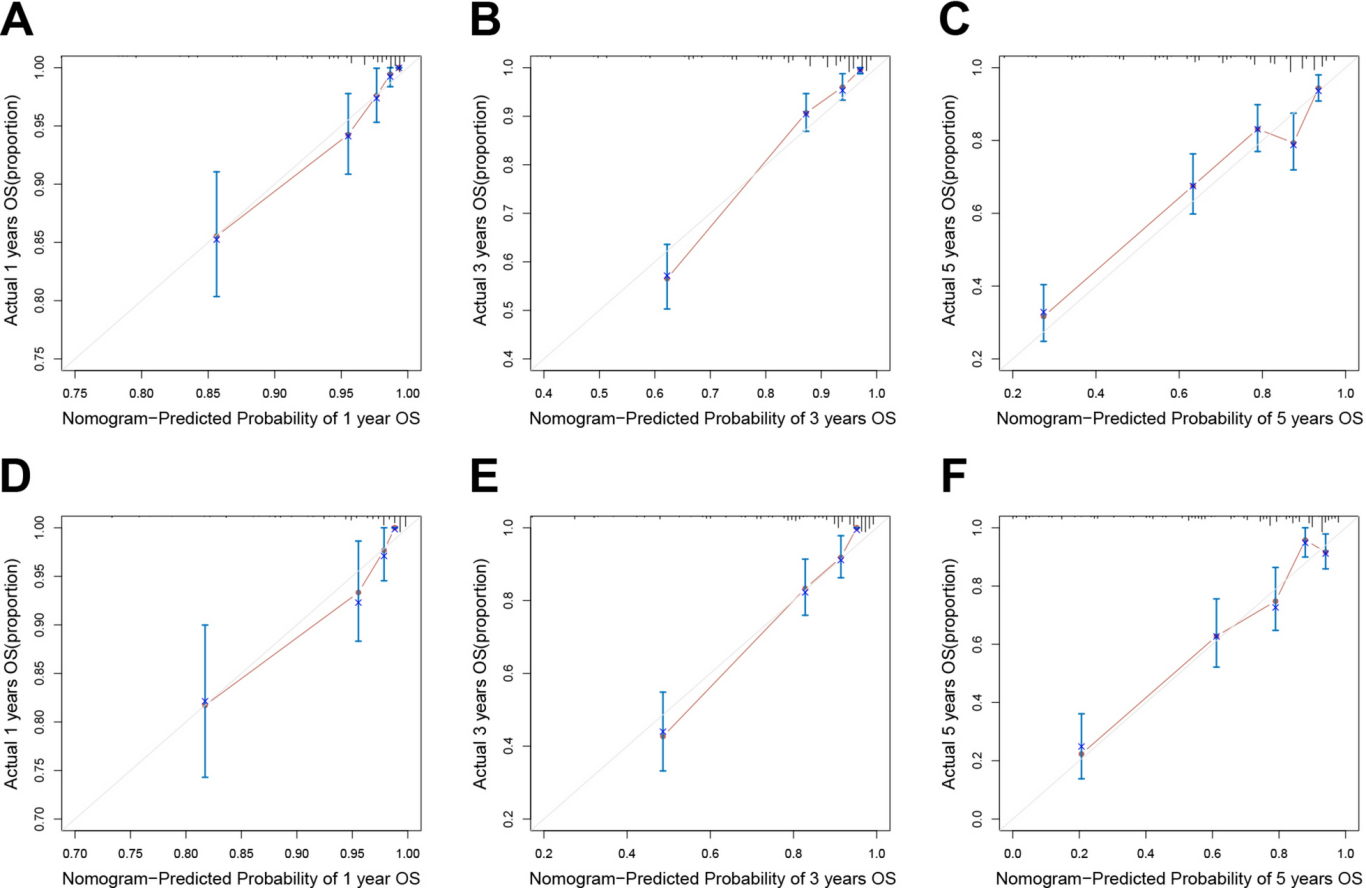
Calibration curve of the nomogram for predicting 1-, 3-, and 5-year OS of colon cancer patients after radical resection in the primary cohort (A, B, C) and validation cohort (D, E, F).

The corresponding C-indices of the model for predicting OS at 1, 3, and 5 years postoperatively were 0.830, 0.859, and 0.757 in the primary cohort, and 0.843, 0.870, and 0.773 in the validation cohort, respectively. The calibration plot of the nomogram, presented in Fig. 4, demonstrates a good consistency between the predicted probability and actual observation in the two cohorts.

**Fig. 5:**
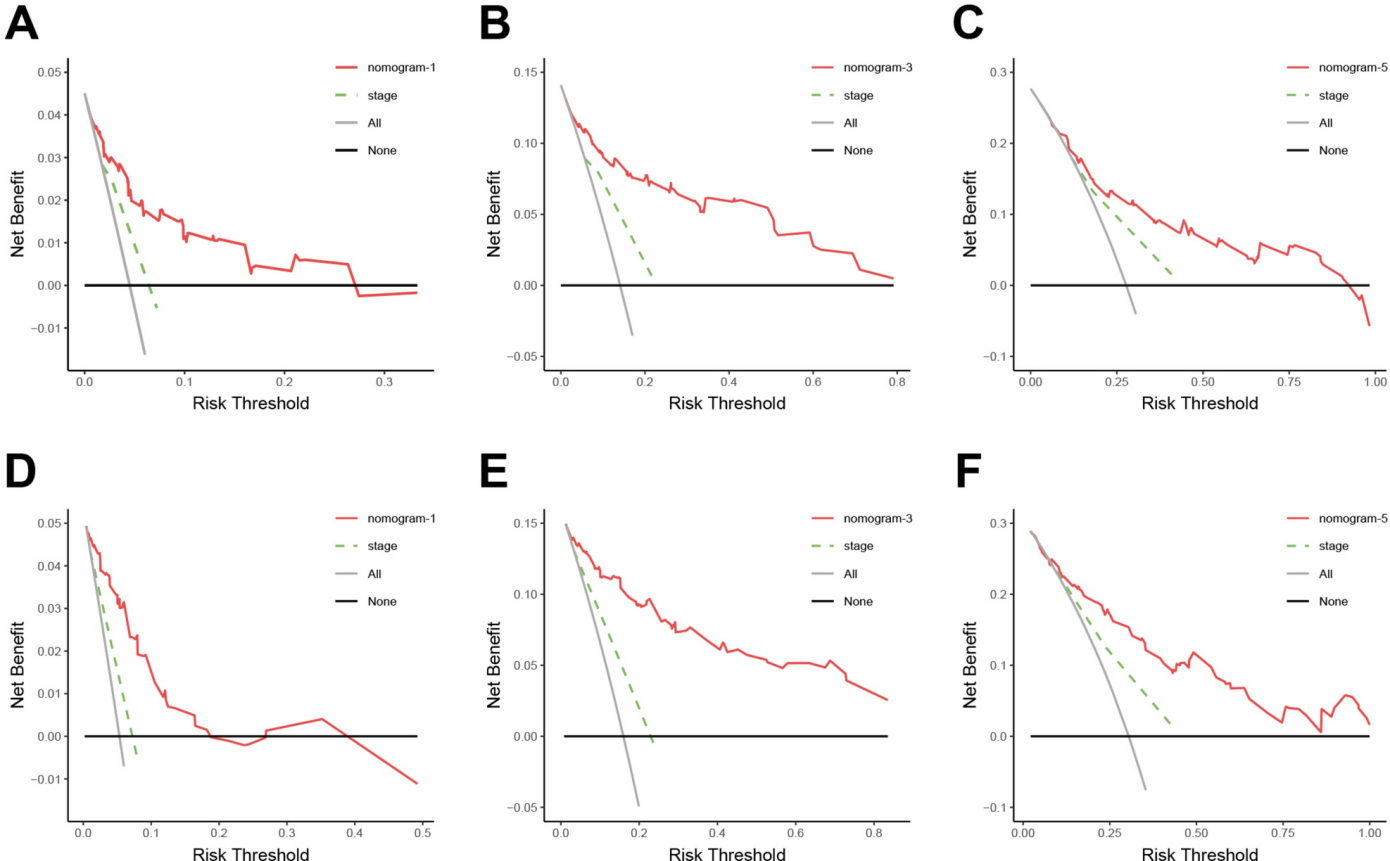
The decision curves of the nomogram to predict 1-, 3-, and 5-year OS in the primary cohort (A, B, C) and validation cohort (D, E, F). Red line: Nomogram model. Dashed green line: TNM staging system model. Grey line: All patients receiving the intervention. Black line: None of the patients receiving the intervention.

To evaluate the application value, a DCA of the nomogram and TNM staging system was performed, as illustrated in Fig. 5. The black horizontal axis indicates that no patients received the intervention, resulting in a net benefit of 0. The gray slanted line represents the situation where all patients received the intervention. Clinical interventions guided by the established nomogram and the TNM staging system demonstrated greater net benefits than all-or-none scheme in predicting 1-, 3-, and 5-year OS. Furthermore, using the nomogram to predict OS added more benefits than the TNM staging system alone across a wide range of threshold probabilities.

## Risk stratification of OS by the nomogram

**Fig. 6:**
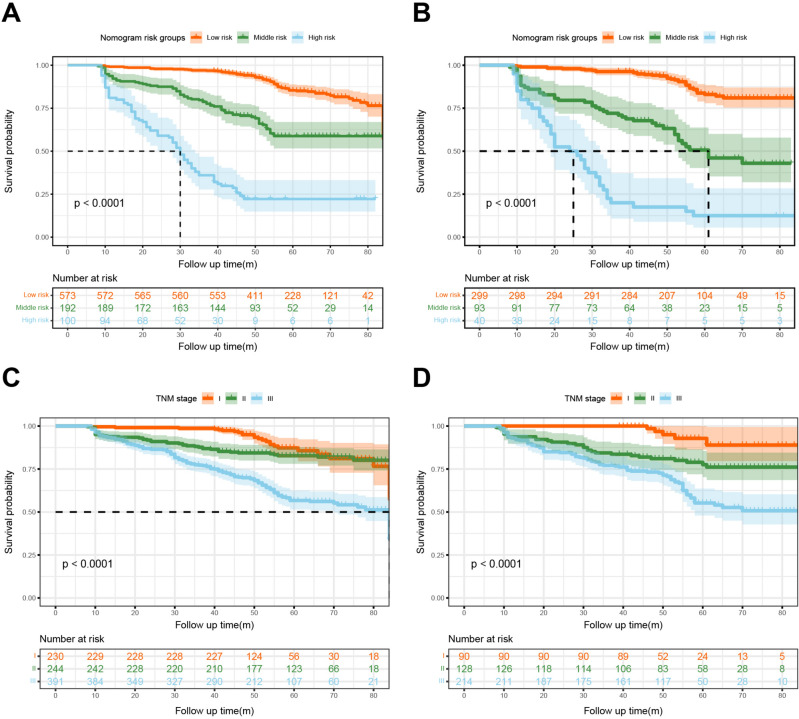
The Kaplan–Meier curves of colon cancer patients following radical resection were stratified by the Nomogram score and TNM staging system in the primary cohort (A, C) and validation cohort (B, D).

To assess the subgroups of patients that were favorably affected by the nomogram, these patients were divided into three groups: low-risk (< 181.30), middle-risk (181.30–259.71), and high-risk (> 259.71) in the two cohorts according to the nomogram score, which indicated significant prognostic classification and outperformed the TNM staging system alone (*P* < 0.0001) (Fig. 6). The mean OS of the three risk groups were 57.85 ± 13.81, 49.12 ± 19.55, and 31.17 ± 17.81 months in the primary cohort and 56.09 ± 13.25, 45.53 ± 21.34, and 30.63 ± 22.55 months in the validation cohort, respectively.

## Discussion

In this retrospective cohort of colon cancer patients, we found that patients who maintained a normal nutritional status were predicted to experience a reduced risk of death and improved survival compared to only considering established clinical and pathologic factors. The prognostic nomogram developed was well calibrated and the corresponding C-index for 1-, 3-, and 5-year OS were respectively 0.830, 0.859, and 0.757 in the primary cohort which was higher than the most of prediction models on survival of colon cancer (C-index ranging from 0.60 to 0.70)^7,16,17^, indicating improvement of modeling performance. Nutritional status elements were associated with more clinical benefits, indicating such improvement to be meaningful. Internal validation in the validation cohort also exhibited excellent discrimination and calibration performance. Hence, utilizing this nomogram could contribute to identifying colon cancer patients with a high risk of mortality and then optimize individualized decision-making regarding cancer management.

Colon cancer is one of the most common malignant tumors in the digestive system, leading to the deaths of thousands annually^1,18^. The possibility of extending survival has been the subject of exploration globally, as improving life is a consistent goal. Currently, the estimation of survival rate in colon cancer patients following radical resection primarily focused on clinical and pathologic characteristics, often overlooking preoperative nutritional status. However, due to the disease’s impact on food intake and nutrient digestion and absorption, patients with colon cancer are at significant risk for malnutrition^9,19^, which can adversely affect their mortality risk. Thus, integrating nutritional status assessments with clinical and pathological features may enhance the reliability of survival predictions for colon cancer patients.

Recently, several studies have demonstrated the significant correlation between nutritional status and clinical outcomes in colon cancer patients. Specifically, the patients with NRS 2002 ≥ 3 were particularly susceptible to postoperative complications, such as anastomotic leakage and wound infection^20,21^. Research involving 506 patients, including those with colon cancer, identified nutritional risk based on NRS 2002 as an independent predictor for mortality over 180 days^13^. Our findings further confirmed the predictive value of preoperative NRS 2002 score on long-term survival of colon cancer patients following radical resection. Additionally, L3MI is considered as an objective indicator of systemic metabolism and nutritional status, with declines in L3MI correlating with poorer clinical outcomes in colon cancer, including increased postoperative complications, shorter disease-free and overall survival^22,23,24^. Nevertheless, a recent study reported no association between L3MI and long-term survival of colon cancer patients undergoing laparoscopic surgery^25^. This discrepancy may be attributed to different operation types and tumor stages. In our study of 1,297 stage I-III colon cancer patients who underwent either laparotomy or laparoscopic surgery, LL3MI, defined by sex-specific cut-off points, demonstrated independent prognostic value for OS. The mechanisms underlying this effect may be associated with hyperinflammatory response, reduced immune function, and insulin resistance, induced by the loss of skeletal muscle mass^26,27,28^.

Preoperative nutritional assessment is essential for cancer patients, as it guides nutritional interventions and improves clinical outcomes^14^. However, due to time constraints, clinicians may not always thoroughly evaluate or recommend appropriate nutritional support, which can lead to poor patient adherence and low prevalence of nutritional interventions in China^29,30^. The NRS 2002 and LL3MI included in our nomogram provide a simple-to-use nutritional evaluation approach that minimizes information collection burdens and enhances clinical applicability^31^. Importantly, the nomogram, which combines NRS 2002 and LL3MI with other significant variables (bowel obstruction, T stage, and N stage), exhibited strong predictive ability for personalized survival estimates in colon cancer patients. This prediction model could facilitate clinicians in identifying patients with an increased risk of cancer death due to poor preoperative nutrition conditions. Consequently, our findings enable clinicians to provide patients with interventions, including dietary advice, the treatment of symptoms impairing food intake, and oral nutritional supplementation, toward optimal nutritional status, thereby preventing cancer progression and improving survival rates.

Currently, The TNM staging system is widely accepted as a prognostic assessment tool for colon cancer, yet it has certain limitations. This system only takes the anatomical extent of disease into account^32^, overlooking other crucial factors such as age, CEA, bowel obstruction, and tumor differentiation. In our study, multivariate Cox analysis identified NRS 2002, LL3MI, bowel obstruction, T stage, and N stage as significant prognostic factors for colon cancer patients. Moreover, clinical decision curve analysis demonstrated that the nomogram based on these significant factors provided more benefits than the TNM staging system alone across all ranges, highlighting the greater clinical utility of our model.

Our study has several advantages. First, to our knowledge, our nomogram is the first predictive tool that simultaneously incorporates preoperative nutritional status and clinical and pathologic characteristics to predict the long-term OS in colon cancer patients after surgery. Second, our study rigorously conformed to the Transparent Reporting of a multivariable prediction model for Individual Prognosis Or Diagnosis (TRIPOD) guidelines^33^. Finally, the prediction model was developed using a large cohort with well-controlled baseline characteristics, theoretically enhancing predictive performance. However, this study is not devoid of limitations. First, being a monocentric study conducted in a single country restricts the generalizability of our results, including the nomogram application to other centers and countries. Multiple-center studies with broader geographic recruitment are needed to validate this nomogram. Second, due to data unavailability, molecular or genetic biomarkers known to have independent prognostic value were not included. But the nomogram can be easily updated and optimized in future study by introducing new variables. Third, we only collected perioperative data, which may not reflect the latest advancements in colon cancer treatment, such as immunotherapy, potentially resulting in an underestimation of survival rates. Even so, our nomogram still demonstrates reliable prognostic value for colon cancer patients and emphasizes the significance of preoperative nutritional interventions in improving survival. Finally, as this investigation is a retrospective study, large-scale prospective randomized controlled trials are warranted to further evaluate its clinical benefit.

## Conclusions

In conclusion, our dynamic online nomogram is a user-friendly tool that utilizes easily accessible baseline data to provide robust estimates of long-term survival in colon cancer patients after radical resection. This tool may help identify patients who would benefit the most from nutrition support and represent an improvement in predicting OS compared to the TNM staging system, potentially facilitating highly personalized cancer management.

## Materials and methods

### Patient selection

Prospective follow-up data of 1297 patients with colon cancer treated at the Affiliated Hospital of Jiangnan University between April 1, 2015 to May 31, 2019 were retrospectively collected. The patients were randomly allocated to the primary and validation cohorts in a 2:1 ratio using R version 4.0.3. The inclusion criteria for both cohorts included: (1) age of 18–60 years, (2) postoperative pathological diagnosis of colon cancer, (3) undergoing radical resection, and (4) TNM stage I to III. The exclusion criteria were as follows: (1) incomplete clinical data, (2) death in the hospital or within 30 days postoperatively, (3) preoperative neoadjuvant therapy, (4) concurrent secondary tumor or rectal cancer, (5) previous history of other malignancies, and (6) pregnancy. This study was approved by the Ethics Committee of the Affiliated Hospital of Jiangnan University (no. 20220025). All methods used in this study adhere to the Declaration of Helsinki. Due to the retrospective nature of the study, the Ethics Committee of the Affiliated Hospital of Jiangnan University waived the requirement for informed consent from all subjects.

## Data collection

Through the prospectively maintained colon cancer database, the following clinical data were carefully collected for each patient: demographics and clinical features [sex, age, body mass index (BMI), age-adjusted Charlson Comorbidity Index (aCCI), eastern cooperative oncology group (ECOG) performance status, carcinoembryonic antigen (CEA), albumin, and bowel obstruction], nutritional status [nutrition risk screening 2002 (NRS 2002), patient-generated subjective global assessment (PG-SGA), third lumbar spine skeletal muscle mass index (L3MI), nutritional risk index (NRI), total protein, hemoglobin, triglycerides, and cholesterol], surgical characteristics [operation type and postoperative comprehensive complication index (CCI)], and pathological information (tumor location, size, differentiation, histology, and stage). These candidate variables of interest were derived from previous studies and clinical experience. Postoperative complications were assessed using CCI scores^34^, where a CCI score of 26.2 was considered the threshold for serious complications^35^. Tumor staging was performed based on pathological reports using the American Joint Committee on Cancer 8th edition.

## Nutritional status assessments

**NRS 2002.**The NRS 2002 was used to evaluate nutritional risk, which accounts for three parts: disease severity (stress metabolism due to the degree of illness), impaired nutritional status (based on weight loss, BMI, and food intake), and 70-year age adjustment (Supplementary Table 2). Patients are scored using the first two components, a score of 0–3 according to whether they are absent, mild, moderate, or severe, and a score of 1 is added if the patient is aged ≥ 70 years. The NRS 2002 score, ranging from 0 to 7, was categorized into three groups: no nutritional risk (0–2), mild nutritional risk (3–4), and severe nutritional risk (≥ 5)^36^.

**PG-SGA**. The PG-SGA, specially designed for assessing nutritional status in cancer patients, comprises four patient self-report components (weight loss, food intake, symptoms, activities and functions) and three professional assessment components (disease and its relationship to nutritional needs, metabolic demand, and physical examination of fat, muscle, and fluid status)^37^. The original PG-SGA and its rating methods are available on the PG-SGA/Pt-Global Platform (https://pt-global.org/pt-global/). We used only the global PG-SGA categories of well nourished (A), moderately malnourished (B), and severely malnourished (C).

**L3MI.**The skeletal muscle index of the third lumbar vertebra (L3MI) is widely used to assess the nutritional status and reflects protein-energy malnutrition^38^. The OsiriX open-source software (version 8.5.2: Pixmeo Sarl, Geneva, Switzerland) was employed to analyze computed tomography images using muscle-specific Hounsfield Unit thresholds of -29 to 150^39^. The cross-sectional areas of the skeletal muscles at L3, including the psoas, erector spinae, quadratus lumborum, transversus abdominis, external and internal obliques, and rectus abdominis muscles (Supplementary Fig. 2) were calculated (cm^2^) and subsequently normalized by the square of the height (m^2^) to obtain the L3MI. According to the previous study, a priori definition of low L3MI (LL3MI) was used (male: L3MI < 40.8 cm^2^/m^2^ and female: L3MI < 34.9 cm^2^/m^2^)^40^.

**NRI.**The NRI, a nutritional evaluation score, was calculated using the formula where NRI = 1.519 × serum albumin (g/L) + 41.7 × (actual body weight [kg] / ideal body weight [kg]). Ideal body weight was determined based on the Lorenz formulas: height (cm) –100 – ([height (cm) – 150] / 4) for men and height (cm) – 100 – ([height (cm) – 150] / 2.5) for women^41^. If the actual body weight was greater than the ideal body weight, the ratio was set at 1 as in previous studies^42,43^. Patients were grouped into previously established categories of non-malnourished (≥ 100), mild (≥ 97.5 to < 100), moderate (≥ 83.5 to < 97.5), or severe malnourishment (< 83.5) according to NRI^43^.

## Study outcome

The primary outcome of our study was OS, defined as the time from the diagnosis of colon cancer to death from any cause. Information on treatment and living status was obtained through telephone, outpatient, or inpatient follow-ups after completion of the primary therapy. Follow-up visits were scheduled every three months for first two years postoperatively and every six months for three years and beyond. The end date of the follow-up was November 30, 2022.

## Statistical analysis

Statistical analyses were performed using R (version 4.0.3; http://www.r-project.org/). The measurement data included in this study were converted to categorical variables, presented as frequencies and corresponding percentages. Chi-squared tests were used to compare the baseline features between the primary and validation cohorts. A univariate Cox regression analysis was performed for each variable, and the significant factors were included in the multivariate Cox regression analysis to identify independent risk predictors associated with OS using the enter method. A nomogram was accordingly formulated based on these significant predictors using R version 4.0.3. For user convenience, an interactive dynamic nomogram was constructed using the package of DynNom. Five-fold cross-validation was performed 1000 times for internal validation of the prediction model. The discrimination performance and predictive accuracy were assessed by the concordance index (C-index), receiver operating characteristic curve (ROC), and calibration curve. A decision curve analysis (DCA) was conducted to assess the clinical benefits of the model. The same assessments were performed in the validation cohort. The total points of each patient were calculated based on the established nomogram model, and three groups of patients with different prognostic risks were depicted using X-tile (version 3.6.1)^44^. Survival curves were delineated by the Kaplan–Meier analysis according to the dichotomized risk groups and compared using the log-rank test. All tests were two-sided, and statistical significance was set at* P* < 0.05.

## Data Availability

Data presented in this study and analytic code are available from the corresponding author upon reasonable request.

## Supplementary Information

Below is the link to the electronic supplementary material.

Supplementary Information.
