# Supplementary material for: Development and validation of a dynamic nomogram for individualized prediction of survival in patients with colon cancer
Source: Sci Rep. 2024 Nov 14;14:28033. doi: 10.1038/s41598-024-78783-8 (PMC11564546; doi:10.1038/s41598-024-78783-8)
Supplement: Supplementary file 1 — Supplementary Information. [file 41598_2024_78783_MOESM1_ESM.pdf]

Supplementary materials

Variables	Univariate analysis		Multivariate analysis	
	HR (95% CI)	*P*	HR (95% CI)	*P*
Sex				
Male vs. Female	1.27(0.87-1.83)	0.212	-	-
Age, years				
≥ 60 vs. < 60	1.23(0.84-1.79)	0.276	-	-
BMI, kg/m^2				
18.5-24 vs.< 18.5	0.69(0.39-1.24)	0.214	-	-
> 24 vs.< 18.5	0.47(0.23-1.05)	0.066	-	-
aCCI				
2-3 vs. 0-1	0.83(0.51-1.35)	0.443	-	-
≥ 4 vs. 0-1	0.96(0.59-1.55)	0.871	-	-
ECOG				
1-2 vs. 0	1.32(0.91-1.92)	0.147	-	-
CEA, μg/L				
≥ 5 vs.< 5	1.26(0.87-1.82)	0.213	-	-
Hypoproteinemia				
Yes vs. No	1.11(0.77-1.61)	0.579	-	-
Bowel obstruction				
Yes vs. No	2.44(1.70-3.50)	< 0.001	2.12 (1.45-3.11)	< 0.001
NRS 2002				
3-4 vs. 0-2	2.52(1.61-3.94)	< 0.001	3.15 (1.97-5.06)	< 0.001
≥ 5 vs. 0-2	4.91(3.07-7.86)	< 0.001	4.48 (2.63-7.61)	< 0.001
PG-SGA				
B vs. A	2.00(1.24-3.23)	0.004	1.08 (0.65-1.81)	0.763
C vs. A	2.90(1.79-4.70)	< 0.001	1.34 (0.98-2.29)	0.074
LL3MI				
Yes vs. No	2.60(1.80-3.76)	< 0.001	2.45 (1.64-3.64)	< 0.001
NRI				
≥97.5 to <100 vs. ≥100	0.82(0.38-1.79)	0.624	-	-
≥83.5 to <97.5 vs. ≥100	0.77(0.42-1.42)	0.403	-	-
<83.5 vs. ≥100	0.81(0.50-1.31)	0.381	-	-
Total protein	1.00(0.98-1.02)	0.746	-	-
Hemoglobin	1.00(0.99-1.02)	0.516	-	-
Triglycerides	0.81(0.58-1.14)	0.223	-	-
Cholesterol	1.01(0.85-1.20)	0.874	-	-
Operation type				
Laparoscopy vs. Laparotomy	0.74(0.47-1.15)	0.174	-	-
CCI ≥ 26.2				

Yes vs. No	1.09(0.69-1.72)	0.711		-	-
Tumor location					
Transverse vs. Right-sided	1.53(0.77-3.05)	0.228		-	-
Left-sided vs. Right-sided	1.28(0.87-1.89)	0.208		-	-
Tumor size, cm					
≥ 5 vs.< 5	1.21(0.84-1.74)	0.311		-	-
Differentiation					
Moderate vs. High	1.12(0.45-2.82)	0.806		-	-
Low vs. High	1.48(0.59-3.68)	0.401		-	-
Histology					
Adeno vs. Non-Adeno	0.68(0.37-1.27)	0.231		-	-
T stage					
T2 vs. T1	1.02(0.38-2.74)	0.970		1.25 (0.45-3.48)	0.675
T3 vs. T1	2.72(1.20-6.17)	0.017		3.71 (1.59-8.65)	0.002
T4 vs. T1	4.11(1.88-8.97)	< 0.001		4.27 (1.85-9.86)	< 0.001
N stage					
N1 vs. N0	1.85(1.15-3.00)	0.012		1.56 (0.94-2.58)	0.086
N2 vs. N0	5.06(3.25-7.87)	< 0.001		3.12 (1.93-5.03)	< 0.001

Table 1. Univariate and multivariate Cox regression analysis of OS in the validation cohort patients. The covariates included in the multivariate analysis were bowel obstruction, NRS-2002, PG-SGA, L3MI, T stage, and N stage. *HR* hazard ratio, *CI* confidence interval.

	Impaired nutritional status	Severity of disease
Absent<br>Score 0	Normal nutritional status	Normal nutritional requirements
Mild<br>Score 1	Weight loss > 5% in 3 months<br>Or<br>Food intake below 50-75% of normal requirement in preceding week	Hip fracture<br>Chronic patients, in particular with acute complications: cirrhosis, COPD<br>Chronic hemodialysis, diabetes, oncology
Moderate<br>Score 2	Weight loss > 5% in 2 months<br>Or<br>Impaired general condition with BMI 18.5-20.5<br>Or<br>Food intake 25-50% of normal requirement in preceding week	Major abdominal surgery<br>Stroke<br>Severe pneumonia, hematologic malignancy
Severe<br>Score 3	Weight loss > 5% in 1 month (≈ > 15% in 3 months)<br>Or<br>Impaired general condition with BMI < 18.5<br>Or<br>Food intake 0-25% of normal requirement in preceding week	Head injury<br>Bone marrow transplantation<br>Intensive care patients
Total score	1. Find score (0-3) for Impaired nutritional status and Severity of disease<br>2. Add the two scores (total score)<br>3. If age ≥ 70 years: add 1 to the total score to correct for frailty of elderly	

Table 2. Nutrition risk screening 2002. *BMI* body mass index; *COPD* chronic obstructive pulmonary disease.

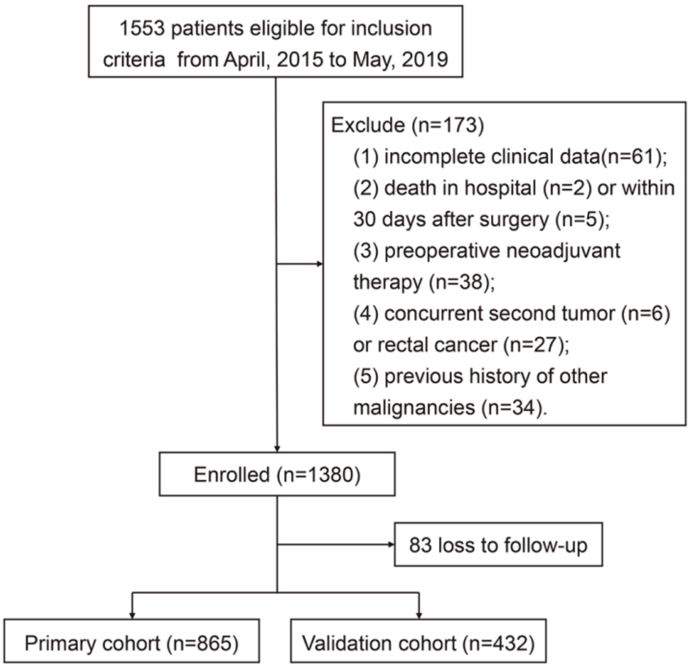

Figure 1. Flow chart of enrolled patients.

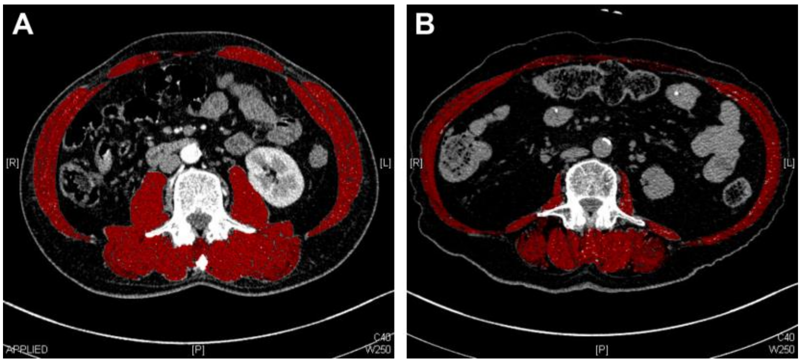

Figure 2. Example of segmentation of skeletal muscle tissue at the third lumbar vertebrae level. The skeletal muscle area was measured by manual tracing in male (A) and female (B).
